# Are Insomnia Type Sleep Problems Associated With a Less Physically Active Lifestyle? A Cross-Sectional Study Among 7,700 Adults From the General Working Population

**DOI:** 10.3389/fpubh.2019.00117

**Published:** 2019-05-14

**Authors:** Rúni Bláfoss, Emil Sundstrup, Markus Due Jakobsen, Hans Bay, Anne Helene Garde, Lars Louis Andersen

**Affiliations:** ^1^National Research Centre for the Working Environment, Copenhagen, Denmark; ^2^Department of Public Health, University of Copenhagen, Copenhagen, Denmark; ^3^Sport Sciences, Department of Health Science and Technology, Aalborg University, Aalborg, Denmark

**Keywords:** sleep initiation and maintenance disorders, sleep wake disorders, exercise, leisure activities, occupational health, epidemiology

## Abstract

**Background:** Sleep problems are common in the general population and negatively affect both private and work life. A vicious circle may exist between poor sleep and an unhealthy lifestyle. For example, poor sleep may drain the energy to do health-promoting physical activity during leisure-time after work. The aim of the present study was to investigate the association between sleep problems and the duration of low- and high-intensity leisure-time physical activity in sedentary and physical workers.

**Methods:** This cross-sectional study employ data from the Danish Work Environment Cohort Study in 2010, where currently employed wage-earners in Denmark on daytime schedule (*N* = 7,706) replied to questions about sleep quality (cf. the Bergen Insomnia Scale) and participation in low- and high-intensity leisure-time physical activity. Associations were modeled using general linear models controlling for various confounders.

**Results:** Workers with high levels of sleep problems reported less high-intensity leisure-time physical activity. Specifically, the weekly duration of high-intensity leisure-time physical activity was 139 (95%CI 111–168), 129 (95%CI 101–158), and 122 (95%CI 92–151) min in sedentary workers with sleep problems < 1, 1–3, and ≥3 days per week, respectively. The same pattern was observed among physical workers. In sedentary workers ≥50 years, the fully adjusted model showed a weekly duration in high-intensity physical activity during leisure of 122 (95%CI 83–161), 102 (95%CI 64–141), and 90 (95%CI 51–130) among those with sleep problems < 1, 1–3, and ≥3 days per week, respectively.

**Conclusions:** Workers, particularly sedentary older workers, having sleep problems report less high-intensity leisure-time physical activity. These data suggest that a vicious circle may indeed exist between poor sleep and reduced leisure-time physical activity.

## Introduction

Sleep problems e.g., difficulties falling asleep, awakening during the night, difficulties awakening and tiredness during the day, are common health complaints among the general adult population with studies reporting a prevalence between 10 and 40% depending on methodologies and definitions of sleep problems (1). Sleep problems are associated with increased risk of cardiovascular diseases (2), obesity and diabetes (3), lost productivity at work (4), and increased risk of workplace injuries (5) resulting in individual suffering and high costs for workplaces (1, 4). Furthermore, sleep problems increase with age (6–9). This may become a larger problem in the near future considering the demographic changes and the increasing retirement age in many Western societies.

Participating in physical activity provides positive effects on general health and prevention of various diseases, e.g., cardiovascular diseases (10). For improving and maintaining health the American College of Sports Medicine recommends to perform moderate-intensity aerobic exercise ≥30 min daily for at least 5 days per week, or vigorous aerobic exercise ≥20 min daily three times per week (10). A positive dose-response association exists between amount of physical activity and health benefits, i.e., the more physically active the better, to a certain point (10). In fact, individuals unable or unwilling to meet the recommendations also benefit from being just slightly more active (10). Moreover, participating in physical activity during leisure can also slow down age-related physiological declines in physical capacity (11). Besides providing positive effects on general health, physical activity also reduces the prevalence of sleep problems (12–15). Thus, a vicious cycle between sleep problems and physical activity may exist, i.e., sleep problems may lead to less physical activity, and less physical activity may worsen sleep problems.

Proper quality sleep and performing regular physical activity is vital for maintaining optimal health. Conversely, sleep problems and poor sleep quality are suggested influencing physical activity levels (16, 17). However, little is known about the associations between sleep problems and the duration of low- and high-intensity leisure-time physical activity, respectively (12). Bromley et al. reported that restricted sleep resulted in a decreased duration and intensity of physical activity (16). The prevalence of sleep problems may vary across work categories and is typically more pronounced in workers with physically demanding job tasks (physical workers) compared with sedentary workers (6, 7). However, even though physically active workers show higher prevalence of sleep problems, participating in physical activity during leisure is associated with lower prevalence of sleep problems in blue-collar workers (18). Thus, physical activity during leisure-time is associated with lower prevalence of sleep problems as well as sleep influences the participation in leisure-time physical activity (12–17).

Based on the above mentioned differences between physical activity at work or during leisure and the associations with sleep problems using both technical (accelerometers) and subjective (questionnaires) methods to examine sleep and physical activity among workers, we wanted to investigate the association between sleep problems and participation in low- and high-intensity physical activity during leisure, both among sedentary and physically active workers. Moreover, because the prevalence of sleep problems is higher with increased age (6–9) and physical capacity declines (11, 19–21), stratified analyses of young and old workers can provide further insight into age-related issues. These analyses are also relevant due to the ongoing demographic changes with an increasing proportion of older workers in many Western societies. Thus, the aim of this cross-sectional study was to investigate the associations between sleep problems (assessed by the Bergen Insomnia Scale) and the duration of low- and high-intensity leisure-time physical activity in young and old as well as sedentary and physical workers. Because sleep problems are more pronounced among physical workers (6, 7), we hypothesized that sleep problems were negatively associated with the duration of both low- and high-intensity physical activity, especially in physical workers.

## Materials and Methods

### Study Design

This cross-sectional study used data from the 2010 round of the Danish Work Environment Cohort Study (DWECS) (22). DWECS contains questionnaires regarding the work environment and health in the general working population of Denmark. The questions used for the present study are specified below. The reporting obeys to the guideline of “Strengthening the Reporting of Observational Studies in Epidemiology” (STROBE) (23).

### Ethics

The present study has been reported to and registered by Datatilsynet (the Danish Data Protection Agency; journal number 2015-57-0074). According to the Danish law, neither approval by ethical and scientific committees, nor informed consent, is needed in questionnaires and register-based studies (24). All the collected data were de-identified and analyzed pseudo-anonymously.

### Participants

The questionnaire was sent to a random sample of 20,000 adult Danish workers aged ≥18 years drawn from the Central Population Register of Denmark (25). A total of 10,605 (~53%) replied (26). The present study included currently employed wage earners on daytime schedule (*N* = 7,706) and thereby excluded shift workers (27), because shift workers experience higher frequencies of sleep problems (28) and the level of leisure-time physical activity may be influenced by levels of light (29, 30). Because not all participants filled in all questions, the exact number of participants for each analysis varies. Demographics and lifestyle characteristics are reported in Table 1.

**Table 1:** Demographics and lifestyle characteristics.

	**Sedentary work**			**Physical work**			
	***N***	**Mean (SD)**	**%**	***N***	**Mean (SD)**	**%**	***P*-value**
Age, years		44.6 (10.5)			44.1 (11.6)		< 0.05
Participants	4,030		52.3	3,676		47.7	
Gender							< 0.001
Male	1,784		44.3	1,792		48.8	
Female	2,246		55.7	1,884		51.3	
BMI							< 0.001
Underweight	29		0.7	22		0.6	
Normal	2,134		54.3	1,767		49.6	
Overweight	1,320		33.6	1,266		35.5	
Obese	445		11.3	508		14.3	
Smoking							< 0.001
No, never	2,075		52.5	1,621		45.2	
Ex-smoker	1,201		30.4	1,024		28.5	
Yes	678		17.2	943		26.3	

*BMI, Body mass index*.

*Significant differences between sedentary work and physical work are provided with p-values*.

### Explanatory Variables

#### Sleep Problems

The prevalence of sleep problems was assessed by the Bergen Insomnia Scale that consists of six questions about sleep problems during the past month and has been validated against subjective and polysomnographic data (31). The main question was “In the past month, how many days on average per week…” with the following response options: (1) “have you spent more than 30 min on falling asleep after turning off the lights,” (2) “have you been awake in a period of more than 30 min throughout the night,” (3) “are you awake more than 30 min earlier than you planned and cannot fall back to sleep,” (4) “have you not felt fully rested after a night sleep,” (5) have you been so tired and sleepy that it affected your work and personal life,” and (6) “have you been dissatisfied with your sleep.” For each of the six sub-questions, the participants responded on a scale of 0–7 days (d) per week. A mean score for the number of days was calculated for the six questions to get an overall score of sleep problems (0–7 d per week). The level of sleep problems was for subsequent analyses divided into “ < 1 d per week”, “1–3 d per week,” and “≥3 d per week.”

#### Physical Activity at Work

Participants were classified as either sedentary workers or physical workers based on their replies to the following question regarding their work: “How would you generally describe your physical activity in your main job?,” with the following response categories (1) “Mostly sedentary work that does not require physical exertion,” (2) “Mostly standing or walking work that otherwise is not physically demanding,” (3) “Standing or walking work with some lifting- and bearing tasks,” or (4) “Heavy or fast work, which is physically demanding” (32). Participants using response category 1 were allocated as sedentary workers, while physical workers were those using response category 2, 3, or 4.

### Outcome Variable

#### Leisure-Time Physical Activity

The duration of leisure-time physical activity among the workers was determined by answering the question “How much time did you on average spend on each of the following leisure-time activities during the past year” using the following sub-questions: (1) “Walking, cycling or other low-intense activity, where you do not get out of breath or sweaty (e.g., Sunday walks, light gardening)?,” (2) “Exercise sports, heavy gardening or fast walk/cycling, where you get sweaty and out of breath?,” and (3) “Vigorous exercise or competitive sports?” (26).

The response categories for each sub-question were:

(1) “≥4 h per week,” (2) “2–4 h per week,” (3) “ < 2 h per week,” or (4) “Do not perform this activity.” For subsequent analyses, the first, second, third and fourth answers were re-coded to be 5, 3, 1, or 0 h weekly for our analyses of duration of leisure-time physical activity (26). Low-intensity leisure-time physical activity was defined as the number of hours spent on the activities from question 1, while high-intensity leisure-time physical activity was defined as the number of hours spent on the activities from question 2 and 3. Afterward, the number of hours was converted to minutes.

### Control Variables

Because sleep quality may be affected by several factors, we adjusted for various potential confounders (33–37). The confounders were age (years, continuous), working hours per week (hours, continuous), body mass index (BMI) (kg/m^2^, continuous), psychosocial work factors [emotional demands and influence at work from the second version of the COPSOQ questionnaire (38)] (continuous scale, 0–100), mental health [from the SF-36 questionnaire (39)] (continuous), gender (“Male,” “Female,” categorical), smoking status (“No, never,” “Ex-smoker,” or “Yes,” categorical) and chronic disease (“Yes,” “No,” categorical) assessed by the question “Has a doctor ever informed you that you have one or more of the following diseases?” with the response options “Yes” and “No” to the following diseases: “depression,” “asthma,” “diabetes (all types),” “cardiovascular disease,” “cancer,” and “back disease.” Participants answering “yes” to one or more diseases were categorized as having chronic disease.

### Statistical Analyses

All statistical analyses in the present study were conducted using the SAS statistical software for Windows (SAS Institute, Cary, NC). We estimated the association between sleep problems (independent variable) and duration of leisure-time physical activity per week (dependent continuous variable) for sedentary workers and physical workers, respectively, using the general linear model's procedure. In the analyses, we also stratified for workers < 50 years and ≥50 years in both sedentary and physical work, i.e., four subgroups. A minimally adjusted model was performed adjusting for age and gender, while a fully adjusted model included all the above-mentioned confounders (age, gender, lifestyle factors, psychosocial work factors, job group, and chronic disease). All potential confounders were included in the statistical models as either continuous or categorical variables as specified in the “Control variables” section. Between-group differences at baseline were analyzed using Student's *T*-test (age) and Chi-Squared test (gender, BMI and smoking). The significance level was set at an alpha level of < 0.05. Unless otherwise stated, results are reported as least square means and differences of least square means (95% confidence limits).

## Results

The percentage of sedentary and physical workers was 52.3 and 47.7%, respectively (Table 1). The percentage of male and female sedentary workers was 44.3 and 55.7%, respectively, while physical worker consisted of 48.8% male workers and 51.3% female workers.

Results are provided in a minimally and a fully adjusted model (Tables 2, 3). In the fully adjusted model for sleep problems and duration of low-intensity leisure-time physical activity (Table 3), younger sedentary workers with sleep problems 1–3 d per week performed 11 min less low-intensity physical activity during leisure (95%CI −20 to −1) compared with workers with sleep problems < 1 d per week. Among physical workers, older workers with sleep problems ≥3 d per week performed 24 min less leisure-time physical activity at low intensity (95%CI −42 to −7).

**Table 2:** Association of sleep problems with low-intensity physical exercise during leisure (minutes per week) among workers with sedentary and physical work, respectively.

				**Sedentary work**				**Physical work**	
**Age-group**	**Sleep problems**	***N***	**%**	**Lsmeans (95% CI)**	**Diff**	***N***	**%**	**Lsmeans (95% CI)**	**Diff**
All^*^	< 1 d per week	1,793	45.5	170 (165 to 174)		1,612	45.2	171 (166 to 176)	
	1–3 d per week	1,579	40.1	162 (157 to 167)	**−8 (−15 to** **−1)**	1,407	39.4	168 (163 to 174)	−3 (−10 to 5)
	≥3 d per week	568	14.4	164 (155 to 172)	−6 (−16 to 3)	548	15.4	159 (150 to 168)	**−12 (−23 to** **−2)**
< 50 yrs^*^	1 d per week	1,133	44.6	170 (164 to 176)		1,006	45.4	165 (158 to 172)	
	1-3 d per week	1,056	41.6	158 (152 to 164)	**−11 (−20 to** **−3)**	897	40.4	163 (156 to 170)	−2 (−11 to 8)
	≥3 d per week	351	13.8	156 (145 to 166)	**−14 (−26 to** **−2)**	315	14.2	166 (154 to 177)	1 (−13 to 14)
≥50 yrs^*^	< 1 d per week	660	47.1	170 (163 to 178)		606	44.9	181 (173 to 190)	
	1–3 d per week	523	37.4	167 (159 to 176)	−3 (−14 to 9)	510	37.8	177 (168 to 186)	−5 (−17 to 7)
	≥3 d per week	217	15.5	177 (164 to 190)	7 (−9 to 22)	233	17.3	150 (137 to 163)	**−31 (−47 to** **−16)**
All^#^	< 1 d per week	1,793	45.5	163 (141 to 184)		1,612	45.2	165 (142 to 189)	
	1–3 d per week	1,579	40.1	152 (131 to 173)	**−10 (−18 to** **−3)**	1,407	39.4	160 (138 to 183)	−5 (−13 to 3)
	≥3 d per week	568	14.4	156 (134 to 178)	−7 (−17 to 4)	548	15.4	158 (135 to 182)	−7 (−19 to 5)
< 50 yrs^#^	< 1 d per week	1,133	44.6	174 (142 to 206)		1,006	45.4	168 (132 to 204)	
	1-3 d per week	1,056	41.6	163 (132 to 195)	**−11 (−20 to** **−1)**	897	40.4	168 (133 to 203)	0 (−11 to 11)
	≥3 d per week	351	13.8	163 (131 to 196)	−10 (−24 to 4)	315	14.2	176 (140 to 212)	8 (−8 to 24)
≥50 yrs^#^	< 1 d per week	660	47.1	162 (131 to 193)		606	44.9	156 (123 to 190)	
	1–3 d per week	523	37.4	151 (120 to 182)	−11 (−24 to 2)	510	37.8	147 (114 to 180)	−10 (−23 to 4)
	≥3 d per week	217	15.5	159 (127 to 191)	−3 (−21 to 14)	233	17.3	132 (98 to 166)	**−24 (−42 to** **−7)**

* *controlled for age and gender*.

# *controlled for age, gender, lifestyle factors (smoking, BMI), work-related factors (physical activity at work, and psychosocial work factors), job group, chronic disease*.

*Significant differences from reference (< 1 d per week) are marked in bold*.

**Table 3:** Association of sleep problems with high-intensity physical exercise during leisure (minutes per week) among workers with sedentary and physical work, respectively.

				**Sedentary work**				**Physical work**	
**Age-group**	**Sleep problems**	***N***	**%**	**Lsmeans (95% CI)**	**Diff**	***N***	**%**	**Lsmeans (95% CI)**	**Diff**
All^*^	< 1 d per week	1,793	45.5	157 (151 to 163)		1,612	45.2	164 (157 to 171)	
	1–3 d per week	1,579	40.1	148 (141 to 155)	−9 (−18 to 0)	1,407	39.4	151 (143 to 158)	–**13 (**–**24 to** –**3)**
	≥3 d per week	568	14.4	139 (128 to 151)	–**18 (**–**31 to** –**5)**	548	15.4	144 (132 –to157)	–**19 (**–**34 to** –**5)**
< 50 yrs^*^	< 1 d per week	1,133	44.6	168 (159 to 176)		1,006	45.4	178 (168 to 188)	
	1–3 d per week	1,056	41.6	157 (149 to 166)	−10 (−22 to 2)	897	40.4	161 (151 to 171)	–**17 (**–**31 to** –**3)**
	≥3 d per week	351	13.8	151 (136 to 166)	−16 (−33 to 1)	315	14.2	157 (140 to 174)	–**21 (**–**41 to** –**2)**
≥50 yrs^*^	< 1 d per week	660	47.1	139 (129 to 148)		606	44.9	142 (131 to 152)	
	1–3 d per week	523	37.4	131 (120 to 141)	−8 (−22 to 6)	510	37.8	134 (122 to 145)	−8 (−23 to 7)
	≥3 d per week	217	15.5	117 (100 to 134)	–**22 (**–**41 to** –**2)**	233	17.3	122 (105 to 139)	−19 (−39 to 0)
All^#^	< 1 d per week	1,793	45.5	139 (111 to 168)		1,612	45.2	157 (125 to 189)	
	1–3 d per week	1,579	40.1	129 (101 to 158)	–**10 (**–**20 to 0)**	1,407	39.4	142 (111 to 173)	–**15 (**–**26 to** –**4)**
	≥3 d per week	568	14.4	122 (92 to 151)	–**18 (**–**32 to** –**3)**	548	15.4	140 (108 to 172)	–**17 (**–**33 to** –**1)**
< 50 yrs^#^	< 1 d per week	1,133	44.6	155 (111 to 199)		1,006	45.4	180 (130 to 231)	
	1–3 d per week	1,056	41.6	147 (104 to 191)	−8 (−21 to 5)	897	40.4	163 (114 to 213)	–**17 (**–**32 to** –**2)**
	≥3 d per week	351	13.8	142 (97 to 187)	−13 (−33 to 6)	315	14.2	163 (112 to 214)	−17 (−40 to 6)
≥50 yrs^#^	< 1 d per week	660	47.1	122 (83 to 161)		606	44.9	110 (68 to 153)	
	1–3 d per week	523	37.4	102 (64 to 141)	–**20 (**–**35 to** –**4)**	510	37.8	99 (57 to 140)	−12 (−29 to 5)
	≥3 d per week	217	15.5	90 (51 to 130)	–**31 (**–**53 to** –**9)**	233	17.3	96 (53 to 139)	−14 (−37 to 8)

* *controlled for age and gender*.

# *controlled for age, gender, lifestyle factors (smoking, BMI), work-related factors (physical activity at work, and psychosocial work factors), job group, chronic disease*.

*Significant differences from reference (< 1 d per week) are marked in bold*.

In the fully adjusted model for sleep problems and high-intensity leisure-time physical activity, older sedentary workers with sleep problems 1–3 d per week and ≥3 d per week performed 20 min (95%CI −35 to −4) and 31 min (95%CI −53 to −9) less high-intensity physical activity during leisure, respectively, compared with workers with sleep problems < 1 d per week. Younger physical workers with sleep problems 1–3 d per week performed 17 min less high-intensity leisure-time physical activity (95%CI −32 to −2) compared with workers with sleep problems < 1 d per week.

The coefficient of determination, *R*^2^, between the different level of sleep problems and duration of leisure-time physical activity among sedentary and physical workers ranged from 0.051 to 0.145.

## Discussion

The main finding in the present study was that sedentary and physical workers with sleep problems performed less high-intensity leisure-time physical activity when adjusted for various potential confounders (age, gender, lifestyle factors, work-related factors, job group, and chronic disease). Most prominently, older sedentary workers with sleep problems 1–3 d per week and ≥3 d per week performed 20 and 31 min less high-intensity physical activity during leisure than older sedentary workers with no sleep problems.

It is important to state that associations between sleep and physical activity may be bi-directional, i.e., sleep may affect physical activity and vice versa in a vicious circle. However, physical exercise elicits beneficial effects on sleep and has been associated with lower prevalence of sleep problems such as disturbed sleep, un-refreshing sleep, satisfaction with sleep and sleep apnea (6, 7, 12–14, 18, 40–42). Opposite, sedentary behavior has been found to associate with higher prevalence of sleep problems (e.g., sleep apnea) (8, 42). Maintaining a moderate physical activity level or increasing leisure-time physical activity level from low to moderate, low to high or moderate to high over a 10 year period reduced the prevalence of self-reported insomnia, i.e., symptoms of disturbed sleep and tiredness during the day (12). In contrast, restricted sleep, 5.5 h per night for 2 weeks, resulted in a decreased amount and intensity of physical activity measured with accelerometers in healthy patients with a parental history of type 2 diabetes (16). However, equivocal evidence exists whether exercise intensity matters for improving sleep. A study found that young adults (mean age: 21 years) adhering to the American College of Sports Medicine's vigorous-intensity exercise recommendations (≥20 min daily three times per week) had better sleep compared with those meeting or exceeding the recommendations for moderate physical activity (43). Furthermore, a prospective study found moderate high-intensity physical activity and vigorous physical activity to elicit beneficial effects on sleep in middle-aged individuals (mean age: 46 years) (44). However, the same study found that only moderate low-intensity physical activity improved sleep in older adults (mean age: 65 years) (44). These findings suggest that exercise intensity plays a certain role depending on the individual's age. Hence, although it is recommended to be physically active at moderate intensity for ≥30 min five times per week, or at vigorous intensity ≥20 min three times per week, both low-, moderate-, and high-intensity physical activity elicit beneficial effects on health and sleep (10, 43, 44). In the present study, the results do not clearly report that workers conforming to the before mentioned guidelines for weekly amount of physical activity for general health had fewer sleep problems, although Table 3 provides some indications. However, in the present study, the two response options to the question about the duration of physical activity during leisure can be interpreted as moderate- and high-intensity, respectively. This makes it difficult to compare our data on the duration of leisure-time physical activity with the guidelines for weekly amount of physical activity (10), because high-intensity (i.e., vigorous activity) in the present study can be a combination of moderate- and high-intensity. However, compelling evidence exists that a physically active lifestyle during leisure, and not at work, protects from experiencing sleep problems (12–15).

A study investigating the prevalence and trends of leisure-time physical activity in U.S. workers found that sedentary workers are more physically active during leisure compared with physical workers (28). Moreover, previous studies have reported a decline in physical activity with age (8, 11, 19–21). Our study elaborates on previous findings showing associations between sleep problems and physical activity level, and that this association is observed among older workers. However, in our study, this association was observed in older sedentary workers. This finding is somewhat surprising, because previous studies have found sedentary workers to be more physically active during leisure (28) and less fatigued after work compared with physical workers (45, 46). Aging is associated with declines in physical capacity resulting in work tasks potentially being performed at relatively higher physical work demands in older workers compared with their younger counterparts (11). However, Cote and co-workers reported that work may be preservative for physical function (47). Because the physical workers in the present study still are working, the results may be biased due to “healthy worker effect.” The physical workers in the present study may, therefore, preserve more energy for participating in high-intensity leisure-time physical activity than the sedentary workers. However, although work may preserve physical capacity (47), physically demanding work does not seem to provide positive effects on health and reduce sleep problems; conversely, physical work seems to increase the prevalence of sleep problems (7). Moreover, based on the physical decline in physical capacity, the demographic changes in many Western societies with an increased proportion of older workers and the definitions of older workers used by agencies, researchers and organizations (48, 49), the present study used the age of 50 years and more as the threshold for being an older worker.

Tables 2, 3 show the importance of controlling for relevant confounders when examining associations between variables. As reported in the present study, besides age and gender, confounders such as lifestyle factors, work-related factors, job group, and chronic disease affect the study-estimates. However, because self-reported data on the duration of physical activity, and especially at low-intensity, is less accurate than e.g., data from accelerometers (50), the clinical relevance of being 11 min less physically active at low-intensity may be practically irrelevant. Particularly in younger workers who need to participate in moderate high-intensity physical activity to acquire positive effects on sleep quality (44). Though, when older physical workers with sleep problems are >20 min less physically active at low intensity during leisure, it may have negative consequences for older workers since moderate low-intensity physical activity has been found to improve sleep in older adults (44). Generally, people perform less physical activity at high-intensity than at low-intensity, since low-intensity comprises walks, gardening etc., while high-intensity, in the present study, comprises activities and exercise associated with heavy breathing and sweating. Based on this, the findings that older sedentary workers with sleep problems 1–3 d per week and ≥3 d per week perform 20 and 31 min less high-intensity leisure-time physical activity, respectively, are of particular interest, since 20 and 31 min equate one session of moderate to vigorous exercise according to the American College of Sports Medicine's recommendations for general health (10). Interestingly, mostly among older sedentary workers the sleep problems were associated with lower levels of high-intensity leisure-time physical activity. Due to the cross-sectional design of the present study, the data do not provide any explanations on this finding. However, according to Tsunoda et al, this may not be an issue, since participation in moderate low-intensity physical activity elicited positive effects on sleep quality in older adults (44). These findings may also reflect reverse causality, i.e., sleep problems cause lower levels of physical activity.

## Strengths and Limitations

The present study contains both strengths and limitations. A strength of the study is the large sample size of 7,706 daytime workers of the general working population stratified for work type (sedentary and physical) and age (< 50 and ≥50 years). Moreover, the sample size of sedentary and physical workers was comparable in regard to number of participants. The large sample size and the comparable size of sedentary and physical workers provide a better foundation in terms of statistical power to compare the four subgroups of the study population. A limitation of the study is the self-reported data on sleep problems and duration and intensity of physical activity. The accuracy of the quantity of sleep problems may be difficult to report, and self-reported levels of leisure-time physical activity have been reported less accurate than e.g., wearing accelerometers to detect activity level (50). Especially low-intensity physical activity seems to be underestimated in self-reports, while high-intensity is more accurate (50). Additionally, the underestimation of low-intensity leisure-time physical activity may be a reason for the small differences in the amount of low- and high-intensity physical activity during leisure (Tables 2, 3). Another reason is that for the workers reporting a physical activity level ≥4 h per week, the hours were re-coded to be 5 h for the data analysis and the reported differences in the present study may, therefore, be quite conservative. Normally, people perform much more low-intense activity than high-intense, since low-intense activity comprises commuting, gardening, walks etc. Therefore, when all hours above 5 h per week are truncated to 5 h, the mean duration of low- and high-intensity leisure-time physical activity may become more equal. Additionally, this method may diminish between-group differences because the highly physically active workers, e.g., 10 h per week, were grouped with workers being physically active 4 h per week. This may have resulted in an underestimation of the duration of both low- and high-intensity leisure-time physical activity among the workers. However, the categorization in the present study was conducted with the purpose to divide the workers into different groups according to their physical activity level, as has been done in previous studies (26, 32). Furthermore, as a limitation, the workers were asked to estimate their weekly duration of leisure-time physical activity during the past year, whereas sleep problems were related to the past month. The results could, therefore, be prone to potential bias, in particular, recall bias. However, as a strength, the sleep questionnaire used was a validated standardized questionnaire to assess sleep problems, and the scale is one of few that is validated against subjective and polysomnographic data (31). Sleep problems can also be classified into sleep onset problems and sleep maintenance problems. It could therefore be a strength to investigate the association between these two type of sleep problems and leisure-time physical activity. Additional analyses were performed on the association between sleep onset problems and sleep maintenance problems with physical activity during leisure (data not shown). These analyses showed an association between sleep maintenance problems and less physical activity during leisure. This was most prominent among older sedentary workers with weekly sleep maintenance problems performing less high-intensity physical activity during leisure. No associations were observed between sleep onset problems and duration of leisure-time physical activity. Though, these analyses were performed by dividing the validated Bergen Insomnia Scale. Therefore, the present study followed the Bergen Insomnia Scale by investigating sleep problems as a sum of six questions about sleep problems (31). A limitation of the present study is that self-reported data may be influenced by common method bias, where e.g., the respondent's mood and socioeconomic status may influence the answers (51). Another limitation is that causal association cannot be inferred by the cross-sectional study design, e.g., sleep problems may lead to less high-intensity physical activity during leisure, but less high-intensity physical activity during leisure may also lead to sleep problems, which again may lead to less high-intensity physical activity. In reality, a vicious cycle between these may exist. However, cross-sectional studies like ours are relatively low cost compared with the huge amount of data generated from a large population. A limitation of the study is that the present study did not assess the incidence of sleep problems, but only provided associations between workers experiencing self-reported sleep problems and duration of physical activity during leisure. As another limitation, the present study did not take timing of physical exercise into account due to lack of data in the dataset. However, conflicting evidence exists on this topic with studies finding that exercise in the late evening may re-establish the circadian rhythm resulting in poorer sleep (52), while other studies have found no negative effects using technical and objective measures to examine the effect of late evening physical exercise on sleep quality among physically fit young adults and non-professional sportsmen (53, 54). A strength of the present study is that we provided a minimally adjusted model (controlling for age and gender) and a fully adjusted model (controlling for age, gender, lifestyle factors, work-related factors, job group, and chronic disease). Clear differences are observed between the minimally and the fully adjusted models, which underscores the importance of controlling for relevant co-variates that can affect the results (33–37). However, controlling for several co-variates can also increase the risk of overadjustment, if some of the confounders function as mediators instead. Another strength of the present study is, that we only included workers on daytime schedule to avoid shift workers, which experience higher frequencies of sleep problems (40). In this cohort study (DWECS 2010), the majority of shift workers are workers with physically demanding works (e.g., nurses). Including these workers would, therefore, increase the risk of biased results in the associations between sleep problems and leisure-time physical activity. Therefore, the generalizability of the present study is confined to the general working population on daytime schedule.

## Conclusions

In the general working population on a daytime schedule, the duration of high-intensity leisure-time physical activity was lower among workers with sleep problems when adjusted for various confounders. This pattern was most pronounced among older sedentary workers. Future studies should investigate the associations between sleep problems and the participation in low- and high-intensity leisure-time physical activity in longitudinal study designs with more accurate and objective measurements.

## Ethics Statement

The present study is a cross-sectional study based on questionnaires and registers. According to the Danish law, neither approval by ethical and scientific committees, nor informed consent, is needed in questionnaires and register-based studies (24). All the collected data were de-identified and analyzed pseudo-anonymously. This has also been stated in the manuscript.

## Author Contributions

RB has written the manuscript. ES has contributed with thorough feedback throughout the whole process. MJ has contributed with thorough feedback throughout the whole process. HB has contributed with his expertise within statistics throughout the process. AG has contributed with her expertise within the field of sleep, and has provided thorough feedback throughout the process. LA has also helped with the statistics and provided thorough feedback and guidance throughout the whole process. All authors interpreted the results, critically revised the manuscript draft and approved the final version of the manuscript for submission.

### Conflict of Interest Statement

The authors declare that the research was conducted in the absence of any commercial or financial relationships that could be construed as a potential conflict of interest.

## References

[1] Léger D Bayon V. Societal costs of insomnia. Sleep Med Rev. (2010) 14:379–89. 10.1016/j.smrv.2010.01.003 20359916

[2] Jacobsen HB Reme SE Sembajwe G Hopcia K Stiles TC Sorensen G. Work stress, sleep deficiency, and predicted 10-year cardiometabolic risk in a female patient care worker population. Am J Ind Med. (2014) 57:940–9. 10.1002/ajim.22340 24809311 PMC4111954

[3] Cappuccio FP D'Elia L Strazzullo P Miller MA. Quantity and quality of sleep and incidence of type 2 diabetes: a systematic review and meta-analysis. Diabetes Care. (2010) 33:414–20. 10.2337/dc09-1124 19910503 PMC2809295

[4] Rosekind MR Gregory KB Mallis MM Brandt SL Seal B Lerner D. The cost of poor sleep: workplace productivity loss and associated costs. J Occup Environ Med. (2010) 52:91–8. 10.1097/JOM.0b013e3181c78c30 20042880

[5] Uehli K Mehta AJ Miedinger D Hug K Schindler C Holsboer-Trachsler E. Sleep problems and work injuries: a systematic review and meta-analysis. Sleep Med. Rev. (2014) 18:61–73. 10.1016/j.smrv.2013.01.004 23702220

[6] Akerstedt T Knutsson A Westerholm P Theorell T Alfredsson L Kecklund G. Sleep disturbances, work stress and work hours: a cross-sectional study. J Psychosom Res. (2002) 53:741–8. 10.1016/S0022-3999(02)00333-1 12217447

[7] Martins AJ Vasconcelos SP Skene DJ Lowden A de Castro Moreno CR. Effects of physical activity at work and life-style on sleep in workers from an Amazonian extractivist reserve. Sleep Sci. (2016) 9:289–94. 10.1016/j.slsci.2016.10.001 28154743 PMC5279928

[8] Vancampfort D Stubbs B Firth J Hagemann N Myin-Germeys I Rintala A. Sedentary behaviour and sleep problems among 42,489 community-dwelling adults in six low- and middle-income countries. J Sleep Res. (2018) 27:e12714. 10.1111/jsr.12714 29851176

[9] Leger D Guilleminault C Dreyfus JP Delahaye C Paillard M. Prevalence of insomnia in a survey of 12,778 adults in France. J Sleep Res. (2000) 9:35–42. 10.1046/j.1365-2869.2000.00178.x 10733687

[10] Garber CE Blissmer B Deschenes MR Franklin BA Lamonte MJ Lee IM. American College of Sports Medicine position stand. Quantity and quality of exercise for developing and maintaining cardiorespiratory, musculoskeletal, and neuromotor fitness in apparently healthy adults: guidance for prescribing exercise. Med Sci Sports Exerc. (2011) 43:1334–59. 10.1249/MSS.0b013e318213fefb 21694556

[11] Kenny GP Yardley JE Martineau L Jay O. Physical work capacity in older adults: implications for the aging worker. Am J Ind Med. (2008) 51:610–25. 10.1002/ajim.20600 18543279

[12] Spörndly-Nees S Åsenlöf P Lindberg E. High or increasing levels of physical activity protect women from future insomnia. Sleep Med. (2017) 32:22–7. 10.1016/j.sleep.2016.03.017 28366337

[13] Dishman RK Sui X Church TS Kline CE Youngstedt SD Blair SN. Decline in cardiorespiratory fitness and odds of incident sleep complaints. Med Sci Sports Exerc. (2015) 47:960–6. 10.1249/MSS.0000000000000506 25207930 PMC4362810

[14] Kline CE Crowley EP Ewing GB Burch JB Blair SN Durstine JL. The effect of exercise training on obstructive sleep apnea and sleep quality: a randomized controlled trial. Sleep. (2011) 34:1631–40. 10.5665/sleep.1422 22131599 PMC3208839

[15] Kredlow MA Capozzoli MC Hearon BA Calkins AW Otto MW. The effects of physical activity on sleep: a meta-analytic review. J Behav Med. (2015) 38:427–49. 10.1007/s10865-015-9617-6 25596964

[16] Bromley LE Booth JN Kilkus JM Imperial JG Penev PD. Sleep restriction decreases the physical activity of adults at risk for type 2 diabetes. Sleep. (2012) 35:977–84. 10.5665/sleep.1964 22754044 PMC3369233

[17] Štefan L Sporiš G Krističević T Knjaz D. Associations between sleep quality and its domains and insufficient physical activity in a large sample of croatian young adults: a cross-sectional study. BMJ Open. (2018) 8:e021902. 10.1136/bmjopen-2018-021902 30007930 PMC6082468

[18] Skarpsno ES Mork PJ Nilsen TIL Jørgensen MB Holtermann A. Objectively measured occupational and leisure-time physical activity: cross-sectional associations with sleep problems. Scand J Work Environ Health. (2017) 44:202–11. 10.5271/sjweh.3688 29094169

[19] Aagaard P Suetta C Caserotti P Magnusson SP Kjaer M. Role of the nervous system in sarcopenia and muscle atrophy with aging: strength training as a countermeasure. Scand J Med Sci Sports. (2010) 20:49–64. 10.1111/j.1600-0838.2009.01084.x 20487503

[20] Bijnen FC Feskens EJ Caspersen CJ Mosterd WL Kromhout D. Age, period, and cohort effects on physical activity among elderly men during 10 years of follow-up: the Zutphen elderly study. J Gerontol Ser A Biol Sci Med Sci. (1998) 53:M235–41. 9597057 10.1093/gerona/53a.3.m235

[21] Westerterp KR. Daily physical activity and ageing. Curr Opin Clin Nutr MetabolCare. (2000) 3:485–8. 10.1097/00075197-200011000-00011 11085835

[22] Burr H Bjorner JB Kristensen TS Tüchsen F Bach E. Trends in the Danish work environment in 1990-2000 and their associations with labor-force changes. Scand J Work Environ Health. (2003) 29:270–9. 12934720 10.5271/sjweh.731

[23] von Elm E Altman DG Egger M Pocock SJ Gøtzsche PC Vandenbroucke JP. The Strengthening the Reporting of Observational Studies in Epidemiology (STROBE) statement: guidelines for reporting observational studies. J Clin Epidemiol. (2008) 61:344–9. 10.1016/j.jclinepi.2007.11.008 18313558

[24] Committee System on Biomedical Research Ethics Guidelines About Notification 2011. (2011). Available online at: http://www.nvk.dk/~/media/NVK/Dokumenter/Vejledning_Engelsk.pdf (accessed May 2, 2019).

[25] Andersen LL Izquierdo M Sundstrup E. Overweight and obesity are progressively associated with lower work ability in the general working population: cross-sectional study among 10,000 adults. Int Arch Occup Environ Health. (2017) 90:779–87. 10.1007/s00420-017-1240-0 28660321

[26] Calatayud J Jakobsen MD Sundstrup E Casaña J Andersen LL. Dose-response association between leisure time physical activity and work ability: cross-sectional study among 3000 workers. Scand J Public Health. (2015) 43:819–24. 10.1177/1403494815600312 26275641

[27] Andersen LL Garde AH. Sleep problems and computer use during work and leisure: cross-sectional study among 7800 adults. Chronobiol Int. (2015) 32:1367–72. 10.3109/07420528.2015.1095202 26539689

[28] Gu JK Charles LE Ma CC Andrew ME Fekedulegn D Hartley TA. Prevalence and trends of leisure-time physical activity by occupation and industry in U.S. workers: the National Health Interview Survey 2004-2014. Ann Epidemiol. (2016) 26:685–92. 10.1016/j.annepidem.2016.08.004 27659584 PMC5109053

[29] Schlafer O Wenzel V Högl B. [Sleep disorders among physicians on shift work]. Anaesthesist. (2014) 63:844–51. 10.1007/s00101-014-2374-z 25213642

[30] Touitou Y Touitou D Reinberg A. Disruption of adolescents' circadian clock: the vicious circle of media use, exposure to light at night, sleep loss and risk behaviors. J Physiol. (2016) 110(Pt B):467–79. 10.1016/j.jphysparis.2017.05.001 28487255

[31] Pallesen S Bjorvatn B Nordhus IH Sivertsen B Hjørnevik M Morin CM. A new scale for measuring insomnia: the Bergen insomnia scale. Percept Mot Skills. (2008) 107:691–706. 10.2466/pms.107.3.691-706 19235401

[32] Bláfoss R Micheletti JK Sundstrup E Jakobsen MD Bay H Andersen LL. Is Fatigue after work a barrier for leisure-time physical activity? cross-sectional study among 10,000 adults from the general working population. Scand J Public Health. (2018) 47:383–91. 10.1177/1403494818765894 29609495

[33] Ohayon MM. Epidemiology of insomnia: what we know and what we still need to learn. Sleep Med Rev. (2002) 6:97–111. 10.1053/smrv.2002.0186 12531146

[34] Edmé JL Facq J Frimat P Vezina M. Relationship between psychosocial factors at work and incidence of perceived health problems in the GERICOTS cohort. Rev Epidemiol Sante Publique. (2011) 59:295–304. 10.1016/j.respe.2011.05.003 21940127

[35] Bayon V Leger D Gomez-Merino D Vecchierini MF Chennaoui M. Sleep debt and obesity. Ann Med. (2014) 46:264–72. 10.3109/07853890.2014.931103 25012962

[36] Burgard SA Ailshire JA. Putting work to bed: stressful experiences on the job and sleep quality. J Health Soc Behav. (2009) 50:476–92. 10.1177/002214650905000407 20099452 PMC3320737

[37] Mehari A Weir NA Gillum RF. Gender and the association of smoking with sleep quantity and quality in american adults. Women Health. (2014) 54:1–14. 10.1080/03630242.2013.858097 24261545

[38] Pejtersen JH Kristensen TS Borg V Bjorner JB The second version of the Copenhagen psychosocial questionnaire. Scand J Public Health. (2010) 38(Suppl. 3):8–24. 10.1177/1403494809349858 21172767

[39] Bjorner JB Kreiner S Ware JE Damsgaard MT Bech P. Differential item functioning in the Danish translation of the SF-36. J Clin Epidemiol. (1998) 51:1189–202. 9817137 10.1016/s0895-4356(98)00111-5

[40] Härmä M Tenkanen L Sjöblom T Alikoski T Heinsalmi P. Combined effects of shift work and life-style on the prevalence of insomnia, sleep deprivation and daytime sleepiness. Scand J Work Environ Health. (1998) 24:300–7. 9754862 10.5271/sjweh.324

[41] Gubelmann C Heinzer R Haba-Rubio J Vollenweider P Marques-Vidal P. Physical activity is associated with higher sleep efficiency in the general population: the CoLaus Study. Sleep. (2018) 41:zsy070. 10.1093/sleep/zsy070 29617980

[42] Kline CE Krafty RT Mulukutla S Hall MH. Associations of sedentary time and moderate-vigorous physical activity with sleep-disordered breathing and polysomnographic sleep in community-dwelling adults. Sleep Breath. (2017) 21:427–34. 10.1007/s11325-016-1434-9 27837376 PMC5400700

[43] Gerber M Brand S Herrmann C Colledge F Holsboer-Trachsler E Pühse U. Increased objectively assessed vigorous-intensity exercise is associated with reduced stress, increased mental health and good objective and subjective sleep in young adults. Physiol Behav. (2014) 135:17–24. 10.1016/j.physbeh.2014.05.047 24905432

[44] Tsunoda K Kitano N Kai Y Uchida K Kuchiki T Okura T. Prospective study of physical activity and sleep in middle-aged and older adults. Am J Prev Med. (2015) 48:662–73. 10.1016/j.amepre.2014.12.006 25891000

[45] Leino-Arjas P Solovieva S Riihimäki H Kirjonen J Telama R. Leisure time physical activity and strenuousness of work as predictors of physical functioning: a 28 year follow up of a cohort of industrial employees. Occup Environ Med. (2004) 61:1032–8. 10.1136/oem.2003.012054 15550611 PMC1740678

[46] Andersen LL Clausen T Persson R Holtermann A. Dose-response relation between perceived physical exertion during healthcare work and risk of long-term sickness absence. Scand J Work Environ Health. (2012) 38:582–9. 10.5271/sjweh.3310 22714069

[47] Cote MP Kenny A Dussetschleger J Farr D Chaurasia A Cherniack M. Reference values for physical performance measures in the aging working population. Hum Factors. (2014) 56:228–42. 10.1177/0018720813518220 24669556

[48] Poscia A Moscato U La Milia DI Milovanovic S Stojanovic J Borghini A. Workplace health promotion for older workers: a systematic literature review. BMC Health Services Res. (2016) 16(Suppl. 5):329. 10.1186/s12913-016-1518-z 27609070 PMC5016729

[49] Ilmarinen JE. Aging workers. Occup Environ Med. (2001) 58:546–52. 10.1136/oem.58.8.546 11452053 PMC1740170

[50] Ara I Aparicio-Ugarriza R Morales-Barco D de Souza WN Mata E González-Gross M. Physical activity assessment in the general population; validated self-report methods. Nutr Hosp. (2015) 31(Suppl. 3):211–18. 10.3305/nh.2015.31.sup3.8768 25719788

[51] Podsakoff PM MacKenzie SB Lee JY Podsakoff NP. Common method biases in behavioral research: a critical review of the literature and recommended remedies. J Appl Psychol. (2003) 88:879–903. 10.1037/0021-9010.88.5.879 14516251

[52] Yamanaka Y Hashimoto S Takasu NN Tanahashi Y Nishide S-Y Honma S. Morning and evening physical exercise differentially regulate the autonomic nervous system during nocturnal sleep in humans. Am J Physiol Regul Integr Comp Physiol. (2015) 309:R1112–21. 10.1152/ajpregu.00127.2015 26333783

[53] Myllymäki T Kyröläinen H Savolainen K Hokka L Jakonen R Juuti T. Effects of vigorous late-night exercise on sleep quality and cardiac autonomic activity. J Sleep Res. (2011) 20:146–53. 10.1111/j.1365-2869.2010.00874.x 20673290

[54] Arias P Madinabeitia-Mancebo E Santiago M Corral-Bergantinos Y Robles-Garcia V Effects of early or late-evening fatiguing physical activity on sleep quality in non-professional sportsmen. J Sports Med Phys Fitness. (2016) 56:597–605. 27285348

